# Association of Triglyceride-Glucose (TyG) Index With Severe Acute Pancreatitis: A Systematic Review and Meta-Analysis

**DOI:** 10.7759/cureus.83795

**Published:** 2025-05-09

**Authors:** Roshan K Mahat, Vedika Rathore, Ravindra Saxena

**Affiliations:** 1 Department of Biochemistry, Dharanidhar Medical College and Hospital, Keonjhar, IND; 2 Department of Biochemistry, Shyam Shah Medical College, Rewa, IND

**Keywords:** acute pancreatitis, prognostic marker, severe acute pancreatitis, systematic review and meta analysis, tyg index

## Abstract

The triglyceride-glucose (TyG) index has emerged as a surrogate marker for insulin resistance and has been implicated in various metabolic and inflammatory diseases. Its potential role in predicting the severity of acute pancreatitis (AP), particularly severe acute pancreatitis (SAP), remains underexplored. This study aims to evaluate the association between the TyG index and SAP through a systematic review and meta-analysis.

We systematically searched PubMed, Scopus, and Europe PMC databases from inception to March 26, 2025. Eligible studies included adult patients with AP stratified by severity (SAP vs. non-SAP) and reported TyG index values. Data extraction and quality assessment using the Newcastle-Ottawa Scale were conducted independently by two reviewers. A random-effects model was used to compute pooled mean differences (MD), with subgroup and sensitivity analyses to explore heterogeneity.

Ten studies comprising 2262 patients were included. The TyG index was significantly higher in SAP patients compared to non-SAP patients (MD = 0.61; 95% CI: 0.46-0.76; p < 0.0001; I² = 79.1%). The index also predicted ICU admission (MD = 0.52) and mortality (MD = 0.71) in AP patients. Subgroup analyses showed consistent findings across study designs and sample sizes, though geographic variability was observed.

The TyG index is significantly associated with the severity of AP and may serve as a reliable, accessible prognostic biomarker for identifying patients at risk of SAP.

## Introduction and background

Acute pancreatitis (AP) is an inflammatory disorder of the pancreas that can manifest as a mild, self-limiting condition or progress to a severe, life-threatening disease characterized by systemic inflammation and multiple organ failure. The precise mechanisms underlying the transition from localized pancreatic injury to systemic inflammation remain inadequately understood [1]. The global incidence of AP is estimated at 34 cases per 100,000 person-years [2,3]. Clinically, AP typically presents with severe abdominal pain, frequently accompanied by nausea, vomiting, and fever [4]. While the majority of AP cases are mild and resolve with supportive care, approximately 15-20% of patients develop severe acute pancreatitis (SAP) - the most critical form of the disease, defined by persistent organ failure lasting more than 48 hours, often involving systemic complications and requiring intensive care support [5]. This subset of patients is at significantly higher risk for morbidity and mortality. The mortality rate of SAP ranges from 9% to 24% [6,7], and may escalate to 47%-69% in patients exhibiting multiple organ dysfunction syndrome [8,9]. Given these substantial risks, the early identification of patients at risk for developing SAP is essential for optimizing clinical management and enhancing patient outcomes.

Insulin resistance (IR), defined by a diminished cellular response to insulin, is a pivotal factor in the pathogenesis of metabolic disorders and significantly disrupts glucose homeostasis [10]. The early identification of IR is crucial for the implementation of timely interventions aimed at preventing associated complications [11]. The hyperinsulinemic-euglycemic glucose clamp technique is widely recognized as the gold standard for the assessment of IR; however, its utility in routine clinical practice is constrained by its complexity, cost, and time-consuming nature [11]. Recently, the triglyceride-glucose (TyG) index has emerged as a robust surrogate marker for IR, exhibiting notable predictive validity for metabolic disorders, including type 2 diabetes mellitus and non-alcoholic fatty liver disease (NAFLD) [12,13]. The TyG index is calculated using the formula: Ln [fasting triglyceride (mg/dL) × fasting glucose (mg/dL)]/2. It is a simple, cost-effective, and non-invasive marker that has shown good correlation with traditional IR measures [14,15]. Due to its ease of use and strong predictive value, the TyG index is gaining clinical relevance as a screening tool for IR-related conditions. Importantly, conditions such as metabolic syndrome, diabetes, dyslipidemia, and NAFLD have been identified as significant risk factors for the development of AP [16,17]. This observation suggests the potential for the TyG index to be related to the severity and progression of AP.

Considering the clinical importance of an easily accessible and cost-effective predictive tool, assessing the relationship between the TyG index and SAP may have significant implications for early risk stratification and patient management. While emerging studies suggest an association between the TyG index and SAP [18-27], no systematic review has comprehensively synthesized and critically evaluated this relationship. This assertion is based on a comprehensive literature search conducted in PubMed, Scopus, and Europe PMC up to March 2025, following Preferred Reporting Items for Systematic Reviews and Meta-Analyses (PRISMA) guidelines. Therefore, the objective of this systematic review and meta-analysis is to assess the association between the TyG index and SAP, and to determine its potential utility as a prognostic marker in clinical practice.

## Review

Materials and methods

This systematic review and meta-analysis were conducted in accordance with the PRISMA guidelines [28]. The protocol for this study was registered with PROSPERO under registration number CRD420251012452 prior to the initiation of the meta-analysis.

Search strategy

An exhaustive electronic database search was conducted utilizing the following databases: PubMed, Scopus, and Europe PMC, covering the period from database inception to March 26, 2025, to identify all pertinent articles. The search terms included: “triglyceride glucose index”, “triglyceride to glucose index”, “triglyceride-glucose index”, “triglycerides glucose index”, “TyG index”, “TyG”, “severe acute pancreatitis”, “SAP”, “severe AP” and “acute pancreatitis”. Boolean operators “AND” and “OR” were strategically employed to optimize the database search results. The detailed search strategy is outlined in Table 1. The search was limited to English-language publications. Initially, titles of all retrieved articles were reviewed for eligibility, followed by a detailed assessment of abstracts and/or full texts of articles deemed potentially relevant. Furthermore, to ensure the thoroughness of our search strategy, reference lists of selected studies and previous reviews were manually examined.

**Table 1:** Search strategies in databases.

Databases	Search Terms	No of Articles Identified
PubMed/Medline	("triglyceride-glucose index"[All Fields] OR "triglyceride to glucose index"[All Fields] OR "triglyceride-glucose index"[All Fields] OR "triglycerides glucose index"[All Fields] OR "TyG index"[All Fields] OR "TyG"[All Fields]) AND ("severe acute pancreatitis"[All Fields] OR "SAP"[All Fields] OR "severe AP"[All Fields] OR "acute pancreatitis"[All Fields])	65
Scopus	TITLE-ABS-KEY ( ( "triglyceride glucose index" OR "triglyceride to glucose index" OR "triglyceride-glucose index" OR "triglycerides glucose index" OR "TyG index" OR "TyG" ) AND ( "severe acute pancreatitis" OR "SAP" OR "severe AP" OR "acute pancreatitis" ) )	20
Europe PMC	(“triglyceride glucose index” OR “triglyceride to glucose index” OR “triglyceride-glucose index” OR “triglycerides glucose index” OR “TyG index” OR “TyG”) AND (“severe acute pancreatitis” OR “SAP” OR “severe AP” OR “acute pancreatitis”)	145

Eligibility criteria

Inclusion Criteria

Two authors independently performed a comprehensive evaluation and selection of relevant studies, following the established criteria based on the PECOS framework: P (Population): patients of AP; E (exposure): SAP patients; C (comparator): non-SAP patients; O (outcome): TyG index and S (study design): observational studies that provide clear and extractable data regarding the TyG index.

Exclusion Criteria

This study implemented specific exclusion criteria, which encompassed preclinical studies (including both in vitro and animal experiments), review articles, conference papers, non-research correspondence, editorials, commentaries, case reports, and investigations focused exclusively on pediatric or pregnant populations. Furthermore, articles not published in English, research articles lacking full-text availability, and studies with incomplete data or ambiguous methodologies for calculating the TyG index were also excluded from consideration.

Study selection and data extraction

Titles and abstracts of the publications were independently screened by two reviewers to identify studies that potentially met the inclusion criteria. The full texts of these candidate studies were subsequently evaluated for eligibility by the same reviewers. Any discrepancies between the reviewers were resolved through discussion among all authors. The following data were extracted from the studies that were included: first author’s last name, publication year, country of study, study design, diagnostic criteria of AP, number of SAP and non-SAP patients, main findings, and other relevant information. Data were independently extracted by two authors, and any discrepancies were resolved through discussion. The authors of the included studies were contacted for further clarification when necessary. Numerical data were extracted from figures using WebPlotDigitizer (version 5.2).

Quality assessment

To evaluate the quality of the included studies, we used the Newcastle-Ottawa Scale (NOS), which focuses on three main domains: selection, comparability, and outcome. Based on the star rating system, studies were classified as low quality (< 5 stars), medium (5-7 stars), or high quality (> 7 stars) [29]. Two independent authors, RKM and VR, conducted the quality assessments. In cases of disagreement, all authors engaged in discussions to resolve the issues.

Data synthesis and statistical analysis

Statistical analyses were conducted using R software (version 4.4.1), employing the “meta” and “metasens” packages. Due to anticipated clinical heterogeneity across included studies, a random-effects model was applied for meta-analysis. In cases where data were reported as median and interquartile range (IQR), we converted these to means and standard deviations (SD) using validated methods using the meta-analysis accelerator [30]. Given that the TyG index values were continuous and reported without units across all included studies, we calculated pooled mean differences (MD) along with 95% confidence intervals (CI) to compare TyG index values between SAP and non-SAP groups. Additionally, the 95% prediction interval (PI) was reported to estimate the expected range of TyG index outcomes in future studies. Statistical heterogeneity was assessed using the I² statistic, with values exceeding 50% indicative of significant heterogeneity. Cochrane’s Q test further evaluated heterogeneity, with p-values below 0.1 considered statistically significant. To explore potential sources of clinical heterogeneity, subgroup analyses were conducted by study design (prospective vs. retrospective), geographical location (China, Egypt, and others, including India and Korea), and sample size (≥200 and <200 participants). A sensitivity analysis was conducted to assess the robustness and stability of the meta-analysis results. Publication bias was visually examined using Doi plots, with asymmetry quantitatively evaluated by the Luis Furuya-Kanamori (LFK) asymmetry index [31]. Interpretation of the LFK index followed standard thresholds: values within ±1 indicated no asymmetry, between ±1 and ±2 signified minor asymmetry, and values greater than ±2 suggested major asymmetry. Unless otherwise specified, statistical significance was set at p < 0.05.

Results

Characteristics of Included Studies

**Figure 1:**
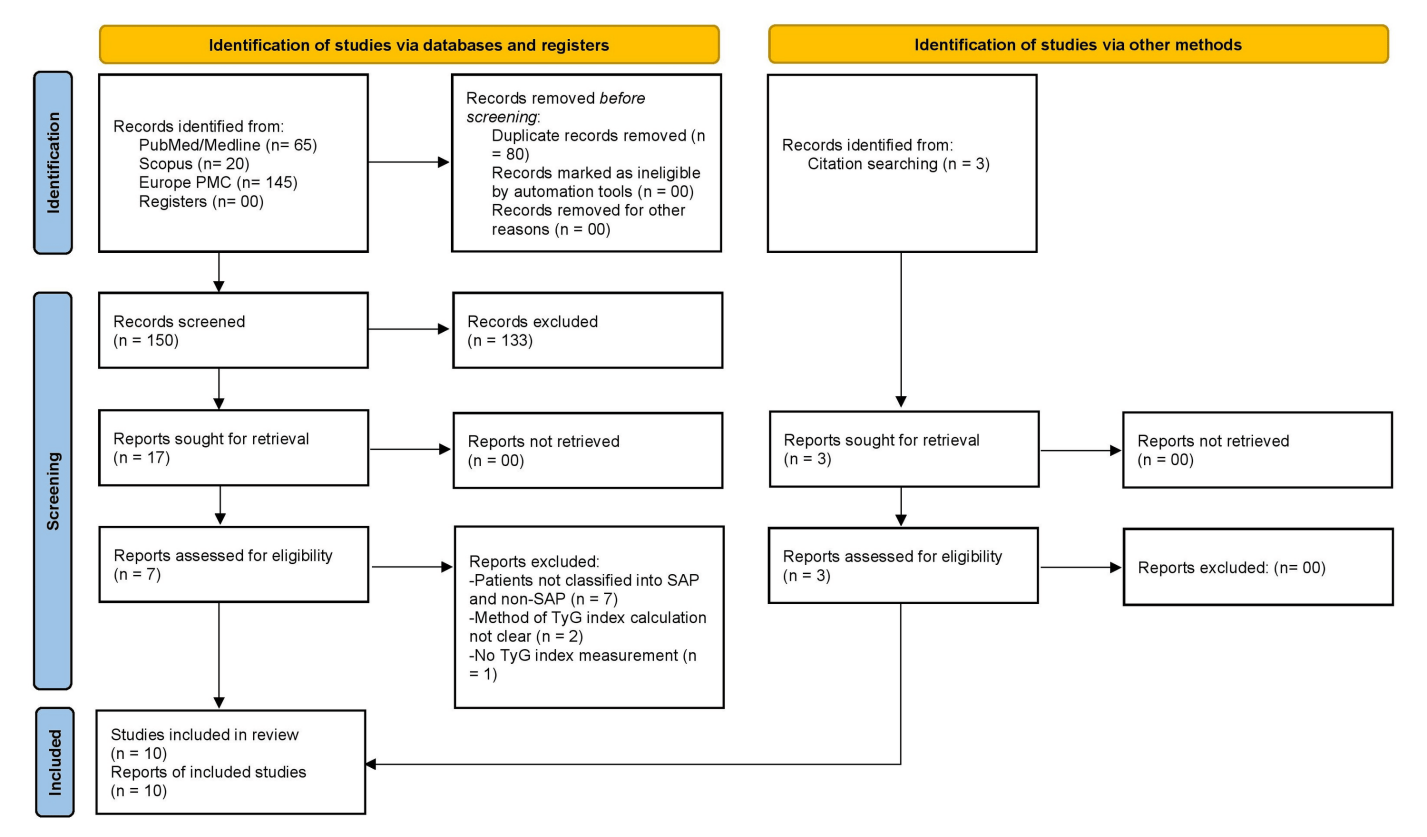
The PRISMA flow diagram for the study selection process. SAP: severe acute pancreatitis

A total of 230 articles were initially retrieved from databases including PubMed (Medline), Scopus, and Europe PMC. Following the removal of duplicates, 150 unique records were screened based on their titles and abstracts, resulting in the exclusion of 133 articles deemed irrelevant. The remaining 17 articles underwent a full-text evaluation, during which 10 were excluded based on the criteria outlined in Figure 1. Additionally, three further studies were identified through citation tracking. Ultimately, 10 studies met all eligibility criteria and were included in the systematic review and meta-analysis [18-27]. The comprehensive study selection process is illustrated in the PRISMA flowchart (Figure 1).

The studies analyzed were published between 2020 and 2025, with six originating from China [20,22,23,25-27], two from Egypt [19,24], one from India [18], and one from the Republic of Korea [21]. Four studies employed a prospective design [18,19,21,24], while the remaining six were retrospective in nature [20,22,23,25-27]. The diagnostic criteria for AP and the severity classification systems utilized across the studies are delineated in Table 2. Furthermore, comprehensive characteristics of all included studies are presented in Table 2.

**Table 2:** Characteristics of the included studies. AP: acute pancreatitis; SAP: severe acute pancreatitis; non-SAP: non-severe acute pancreatitis; MAP: mild acute pancreatitis; MSAP: moderately severe acute pancreatitis; TyG index: triglyceride-glucose index; ICU: intensive care unit; ERCP: endoscopic retrograde cholangiopancreatography; NR: not reported; CT: computerized tomography; MRI: magnetic resonance imaging; ULN: upper limit of normal

Authors	Year	Country	Type of Study	Study Population	Diagnostic Criteria	Severity Classification	Etiologies	Main Findings
Balamurli et al. [18]	2023	India	Single-center, prospective study	Total = 67, SAP = 11, Non-SAP = 56, male = 46, female = 21	AP was diagnosed based on the presence of two of the following three features: (1) typical abdominal pain, (2) serum amylase and/or lipase ≥3 times the upper normal limit, and (3) radiologic findings.	The severity of AP was classified as per the revised Atlanta 2012 criteria.	The most common etiology was gallstone, followed by alcohol consumption and hypertriglyceridemia	The TyG index scores were significantly elevated in the SAP group compared to the non-SAP group. Similarly, patients who required ICU admission had higher TyG index values than those who were not admitted to the ICU. Moreover, individuals who died from AP-related complications exhibited higher TyG index scores than those who survived.
Gadallah et al. [19]	2023	Egypt	Single-center, prospective study	Total = 60, SAP = 17, Non-SAP = 43, Male = 32, Female = 28	The individual presents with persistent upper abdominal pain, accompanied by serum amylase and/or lipase levels elevated to more than three times the upper limit of normal. In addition, imaging findings are consistent with a potential diagnosis of AP.	The severity of AP was classified as per the revised Atlanta 2012 criteria.	Gallstone (35%), hypertriglyceridemia (30%), hypercalcemia (5%), post-ERCP (30%)	The TyG index was significantly elevated in the SAP group compared to the non-SAP group.
Li et al. [20]	2024	China	Single-center, retrospective study	Total = 253, SAP = 60 non-SAP = 193, male = 162, female = 91	NR	The patients were classified into SAP and N-SAP groups according to the revised Atlanta criteria and Ranson and BISAP scoring systems.	NR	The TyG index was significantly higher in the SAP group than in the non-SAP group.
Park et al. [21]	2020	Korea	Multi-center, prospective study	Total = 373, SAP = 25, non-SAP = 348, male: 259, female: 114	AP was diagnosed based on the presence of two of the following three features: (1) typical abdominal pain, (2) serum amylase and/or lipase ≥3 times the upper normal limit, and (3) radiologic findings.	The severity of AP was classified as per the revised Atlanta 2012 criteria.	Gallstones (51.5%), alcohol consumption (38.6%), and hypertriglyceridemia (9.9%)	The TyG index is significantly associated with SAP in patients with acute pancreatitis (AP). Additionally, TyG index scores were higher in patients who required ICU admission compared to those who were not admitted, as well as in patients who died from AP-related complications compared to those who survived.
Qin et al. [22]	2024	China	Single-center, retrospective study	Total = 240, SAP = 72, non-SAP = 168, male = 142, female = 98	The diagnostic criteria for AP were in line with the 2021 Chinese Guidelines for Diagnosis and Treatment of AP: (1) persistent epigastric pain; (2) serum amylase and/or lipase concentrations three times higher than the normal upper limit; (3) abdominal imaging findings were consistent with AP. If two of the above three criteria are met, AP is diagnosed.	The severity of AP was classified as per the revised Atlanta 2012 criteria.	Hypertriglyceridemia (21.67%), gallstone (40.83%), alcoholic (17.5%), others (20%)	The TyG index was significantly higher in the SAP group than in the non-SAP group. In comparison with the survival group, the dead patients had high levels of the TyG index.
Sayiti et al. [23]	2023	China	Single-center, retrospective study	Total = 404, SAP = 59, non-SAP = 345, male = 205, female = 199	The inclusion criteria for AP were based on the presence of two of the following 3 characteristics: (1) typical abdominal pain, and the time from abdominal pain to admission should not exceed 48 hours. (2) serum amylase and/or lipase ≥ 3 times the upper normal limit, and (3) radiologic findings.	The severity of AP was classified as per the revised Atlanta 2012 criteria.	Gallstone (58.9%), hypertriglyceridemia (24%), alcohol (12.6%), idiopathic pancreatitis (4.5%)	The TyG index scores were significantly higher in the SAP group, the ICU-admitted group, and the group of patients who subsequently died, compared to the non-SAP group, those not admitted to the ICU, and the survivors, respectively.
Sieddek et al. [24]	2024	Egypt	Single-center, prospective study	Total = 54, SAP = 7, non-SAP = 47, male = 32, female = 22	AP was clinically diagnosed with two of the following 3 criteria: (1) symptoms (e.g., epigastric pain), (2) a serum amylase or lipase level more than three times the laboratory’s ULN, and (3) CT or MRI imaging consistent with pancreatitis.	The patients were categorized into two groups based on the BISAP score.	gall stone (59.26%), hypertriglyceridemia (29.63%), post ERCP (11.11)	The TyG index was significantly higher in the severe group compared to the non-severe AP.
Wang et al. [25]	2025	China	Single-center, retrospective study	Total = 321, SAP = 71 MAP = 203 MSAP= 47, gender= NA	The inclusion criteria were in accordance with the Chinese Guidelines for Diagnosis and Treatment of Acute Pancreatitis (2019)	According to the criteria outlined in the Chinese Guidelines for Diagnosis and Treatment of Acute Pancreatitis (2019), patients with AP were grouped based on their clinical manifestations and severity.	All were hypertriglyceridemic patients.	TyG index values of the SAP group were higher than those of the MSAP group and the MAP group.
Xinyu et al. [26]	2025	China	Single-center, retrospective study	Total= 137, SAP= 57, MAP= 52, MSAP= 28, male= 91, female= 46	The inclusion criteria were: (1) onset within 48 h, (2) serum amylase or lipase levels exceeding three times the upper normal limit, (3) typical abdominal pain symptoms meeting the diagnostic criteria for AP, and (4) imaging findings indicative of AP. Patients were included if they met at least two of the above criteria, as defined by the 2012 Revised Atlanta Classification.	The severity of AP was classified as per the revised Atlanta 2012 criteria.	Gallstone (35%), alcohol (20.4%), hypertriglyceridemia (17.5%), idiopathic (16.8%), Others (10.2%)	The TyG index increases significantly with the severity of AP.
Yimin and Jianqiang [27]	2022	China	Single-center, retrospective study	Total = 353, SAP = 47, non-SAP = 306, male = 247, female = 106	AP was diagnosed when two of the following three characteristics were present: (1) typical abdominal pain, (2) serum amylase or lipase levels exceeding three times the upper limit, and (3) characteristic findings on ultrasonography (US), CT, or MRI.	The severity of AP was classified as per the revised Atlanta 2012 criteria.	Hypertriglyceridemia (28.6%), gallstone (37.7%), alcoholic (13.9%), others (19.8%)	Compared with the non-SAP group, the TyG index was significantly higher in the SAP group. A high TyG index is closely related to SAP and AP-related complications.

The quality of the included studies ranged from moderate to high, as evaluated using the NOS. Detailed results of the quality assessment are presented in Table 3.

**Table 3:** Methodological quality (risk of bias) assessed by Newcastle-Ottawa Scale (NOS).

Study	Selection	Comparability	Outcome	Total score
Balamurli et al. [18]	***	*	**	6
Gadallah et al. [19]	***	*	**	6
Li et al. [20]	****	**	**	8
Park et al. [21]	****	**	***	9
Qin et al. [22]	***	**	**	7
Sayiti et al. [23]	**	*	**	5
Sieddek et al. [24]	**	*	**	5
Wang et al. [25]	***	**	**	7
Xinyu et al. [26]	***	**	**	7
Yimin and Jianqiang [27]	***	**	**	7

Pooled Analysis of TyG Index in SAP vs. non-SAP

**Figure 2:**
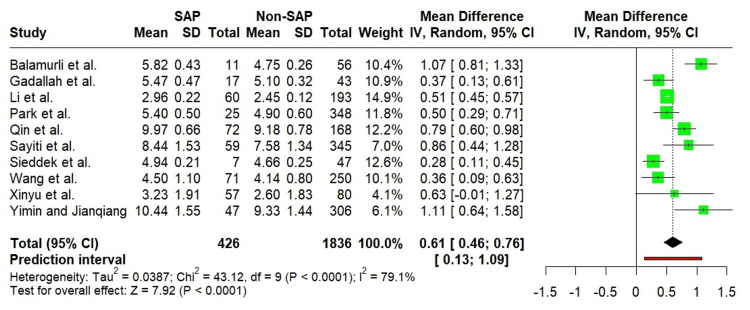
Forest plot showing comparison of the TyG index between SAP and non-SAP. Balamurli et al. [18], Gadallah et al. [19], Li et al. [20], Park et al. [21], Qin et al. [22], Sayiti et al. [23], Sieddek et al. [24], Wang et al. [25], Xinyu et al. [26], Yimin and Jianqiang [27]. SAP: Severe acute pancreatitis; non-SAP: Non-severe acute pancreatitis.

Random-effects meta-analysis of the 10 included studies [18-27] revealed a significantly elevated TyG index in patients with SAP compared to those with non-SAP (MD = 0.61; 95% CI: 0.46 to 0.76; p < 0.0001; I² = 79.1%). The 95% PI indicated that future study outcomes are likely to range between 0.13 and 1.09. Figure 2 provides a forest plot illustrating the comparative analysis of the TyG index between SAP and non-SAP groups.

**Figure 3:**
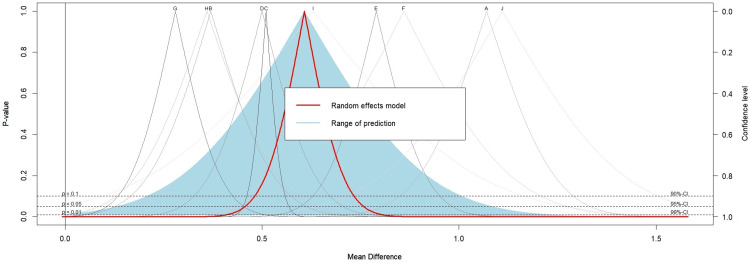
Drapery plot of meta-analysis. The gray curves represent individual primary studies, with their weights in the random-effects model represented on a grayscale. Higher-precision studies are indicated in dark gray, while lower-precision studies appear in light gray. The point estimate for each study is located at the peak of its respective curve. The thick line (red) indicates the pooled effect estimated by the random-effects model. The shaded area (light blue) in the plot represents the prediction interval. Horizontal dashed lines can be used to indicate confidence or prediction intervals for common alpha levels (0.1, 0.05, 0.01). A: Balamurli et al. [18]; B: Gadallah et al. [19]; C: Li et al. [20]; D: Park et al. [21]; E: Qin et al. [22]; F: Sayiti et al. [23]; G: Sieddek et al. [24]; H: Wang et al. [25]; I: Xinyu et al. [26]; J: Yimin and Jianqiang [27].

Additionally, a drapery plot was generated to visually summarize the meta-analysis results through individual study p-value functions, plotting p-values against effect sizes (Figure 3).

Pooled Analysis of TyG Index in Survivors vs. Non-survivors and ICU vs. Non-ICU Patients

**Figure 4:**
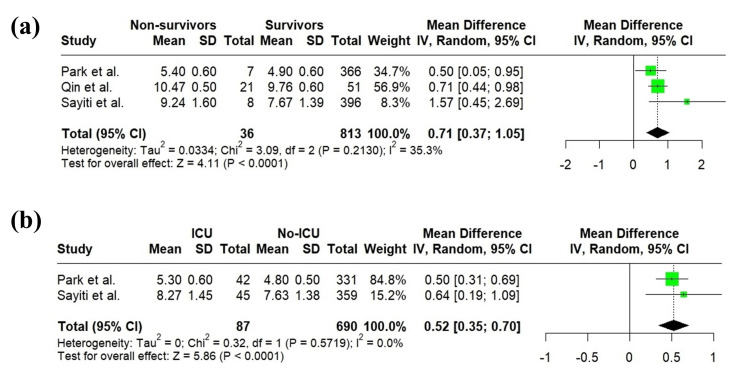
(a) Forest plot showing comparison of the TyG index between survivors and non-survivors. (b) Forest plot comparing the TyG index between patients admitted to the ICU and those not admitted. Park et al. [21], Qin et al. [22], Sayiti et al. [23]

Three studies [21-23] compared the TyG index between survivors and non-survivors. A random-effects meta-analysis showed that the TyG index was significantly higher in non-survivors than in survivors (MD = 0.71; 95% CI: 0.37 to 1.05; p < 0.0001; I² = 35.3%) (Figure 4a). Additionally, two studies [21,23] compared the TyG index between patients admitted to the ICU and those not admitted. The random-effects meta-analysis demonstrated a significantly higher TyG index in ICU-admitted patients compared to non-ICU patients (MD = 0.52; 95% CI: 0.35 to 0.70; p < 0.0001; I² = 0%) (Figure 4b).

Subgroup analysis

**Figure 5:**
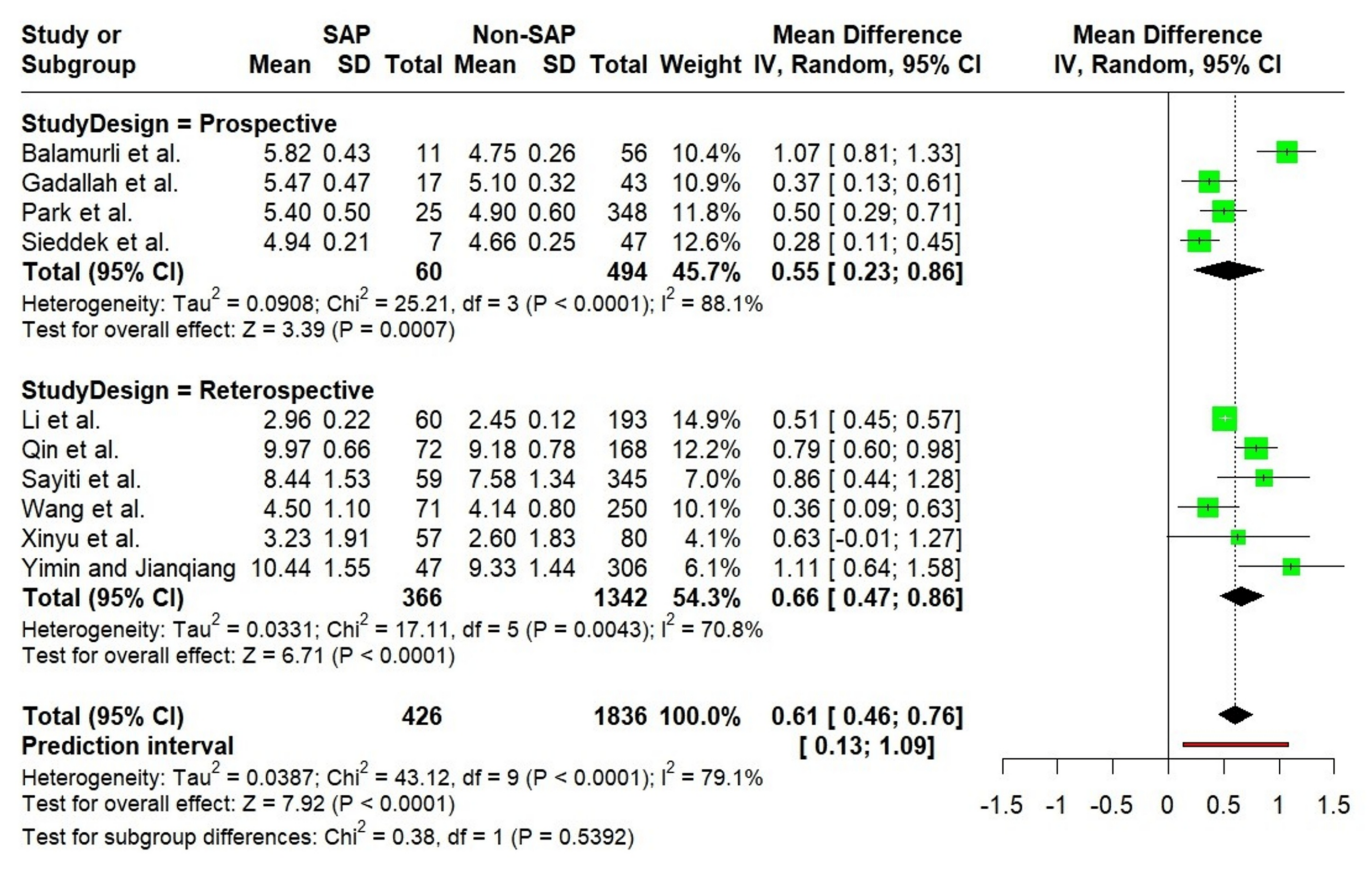
Forest plot showing the subgroup analysis of the TyG index by study design. Balamurli et al. [18], Gadallah et al. [19], Li et al. [20], Park et al. [21], Qin et al. [22], Sayiti et al. [23], Sieddek et al. [24], Wang et al. [25], Xinyu et al. [26], Yimin and Jianqiang [27].

In light of the substantial heterogeneity observed, subgroup analyses were conducted based on study design (prospective vs. retrospective), sample size (<200 vs. ≥200), and geographic region (China, Egypt, and others, including India and Korea). Stratification by study design consistently demonstrated a significantly higher TyG index in SAP compared to non-SAP; however, no statistically significant differences in effect size were noted between prospective and retrospective studies (Figure 5).

**Figure 6:**
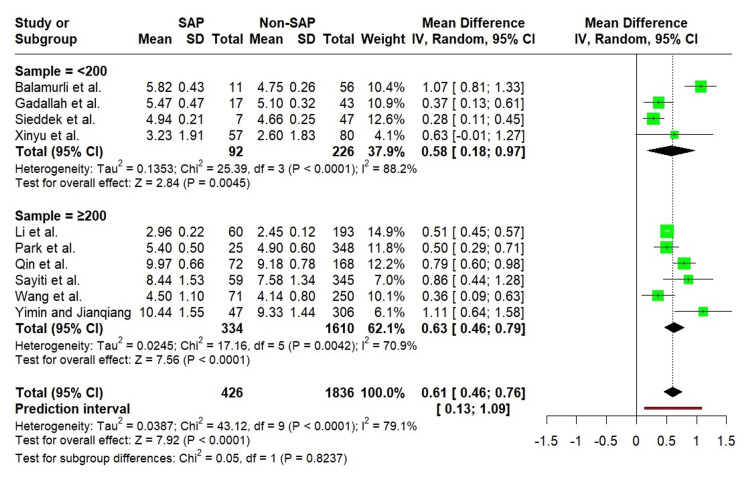
Forest plot showing the subgroup analysis of the TyG index by sample size. Balamurli et al. [18], Gadallah et al. [19], Li et al. [20], Park et al. [21], Qin et al. [22], Sayiti et al. [23], Sieddek et al. [24], Wang et al. [25], Xinyu et al. [26], Yimin and Jianqiang [27].

Similarly, both sample size subgroups (<200 and ≥200) exhibited a significantly elevated TyG index in SAP, without meaningful differences between subgroups (Figure 6).

**Figure 7:**
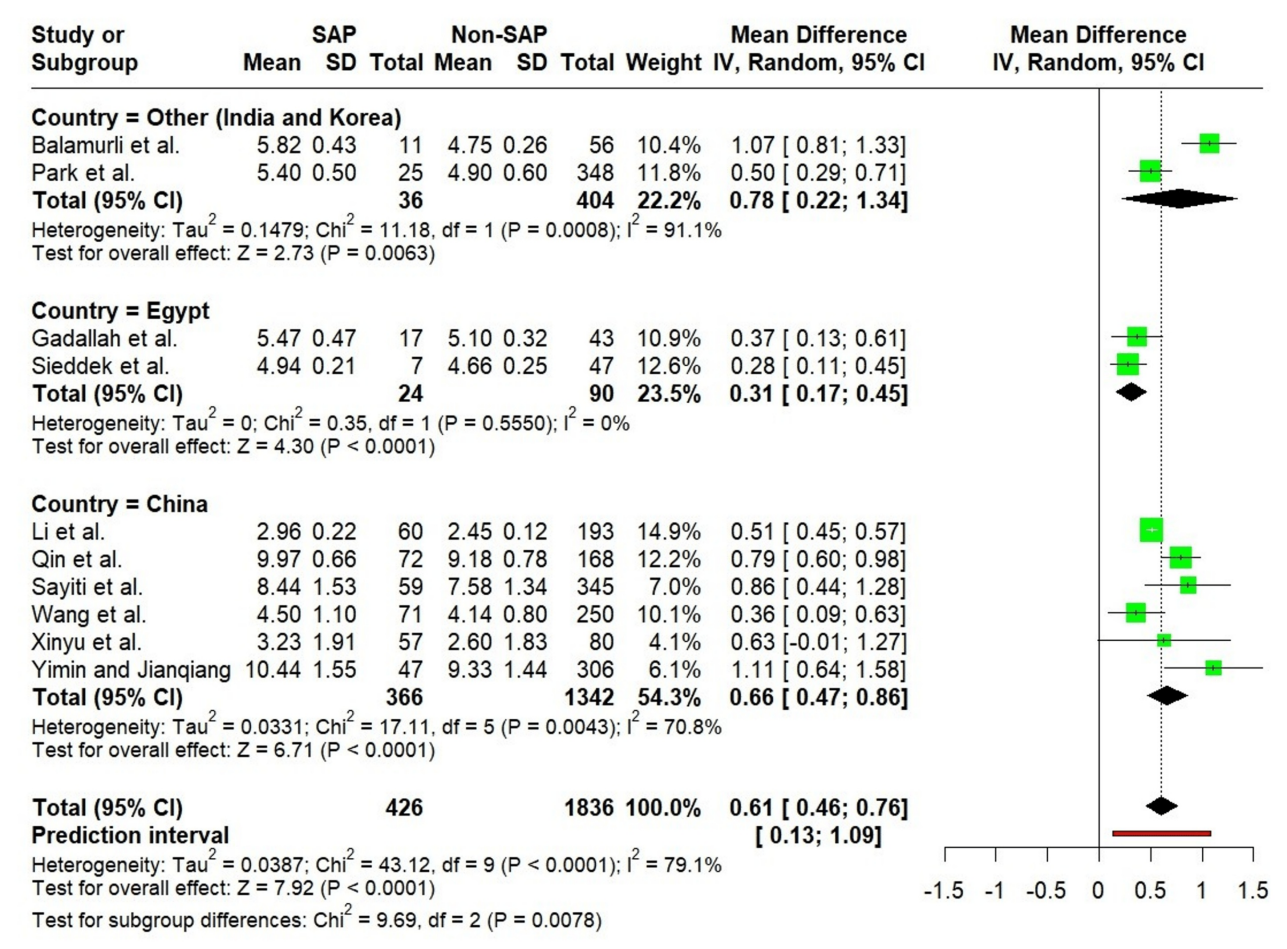
Forest plot showing the subgroup analysis of the TyG index by geographic region. Balamurli et al. [18], Gadallah et al. [19], Li et al. [20], Park et al. [21], Qin et al. [22], Sayiti et al. [23], Sieddek et al. [24], Wang et al. [25], Xinyu et al. [26], Yimin and Jianqiang [27].

When stratified by country, the TyG index was significantly higher in SAP patients across all regional subgroups. Notably, the test for subgroup differences reached statistical significance, indicating that the magnitude of association varied across countries. Among these, studies conducted in Egypt showed no heterogeneity (I² = 0%), suggesting greater consistency in effect estimates within this subgroup (Figure 7).

Sensitivity analysis and publication bias

**Figure 8:**
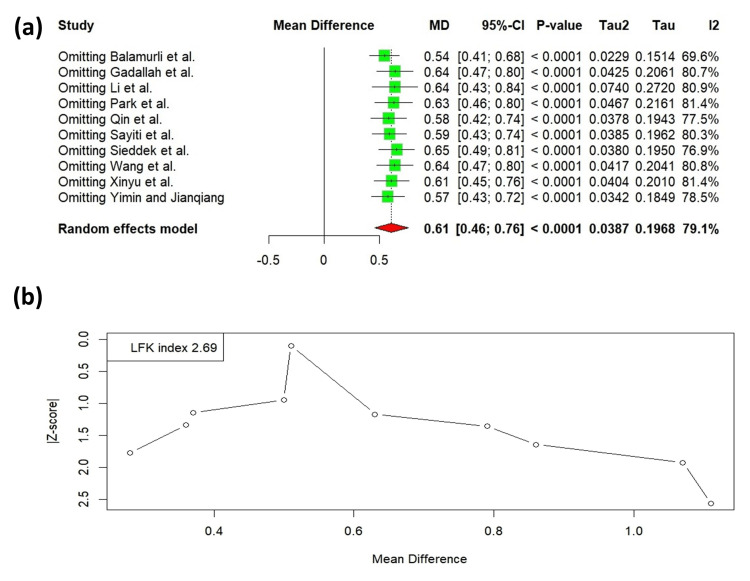
(a) Results of the leave-one-out method in sensitivity analysis for the TyG index. (b) Doi plot showing publication bias for the TyG index. Balamurli et al. [18], Gadallah et al. [19], Li et al. [20], Park et al. [21], Qin et al. [22], Sayiti et al. [23], Sieddek et al. [24], Wang et al. [25], Xinyu et al. [26], Yimin and Jianqiang [27].

Sensitivity analyses were conducted by sequentially excluding individual studies to evaluate the robustness of the findings. The I² values ranged from 69.6% to 81.4%, indicating that no single study was solely accountable for the observed heterogeneity (Figure 8a). An assessment of publication bias utilizing the Doi plot revealed considerable asymmetry, as evidenced by an LFK index of 2.69, which is consistent with the presence of significant asymmetry (Figure 8b).

Diagnostic accuracy of the TyG index for predicting SAP

Table 4 summarizes the diagnostic performance of the TyG index across eight studies [18,19,21-26] in predicting the severity of AP. Reported AUC values ranged from 0.612 to 0.985, indicating variable diagnostic utility. Balamurli et al. [18] demonstrated an exceptionally high discriminative ability (AUC = 0.985; 95% CI: 0.960-1.000; p = 0.0005) with an optimal cutoff value of 5.39, although sensitivity and specificity were not reported. Similarly, Qin et al. [22] and Sieddek et al. [24] also reported robust AUC values (0.826 and 0.827, respectively), with respective sensitivity/specificity values of 72.22%/82.74% and 71.4%/85.1%. In contrast, lower diagnostic performance was observed in studies such as Wang et al. (AUC = 0.612) [25] and Xinyu et al. (AUC = 0.637) [26]. Optimal cutoff values varied widely across studies, ranging from 1.003 to 7.74. Sensitivity ranged from 43.1% to 100%, while specificity varied between 21.2% and 85.1%, reflecting heterogeneity in patient populations and diagnostic thresholds. Notably, Park et al. [21] reported a high sensitivity (92%) but low specificity (37.4%) at a cutoff of 4.92, suggesting its utility in screening settings.

**Table 4:** Diagnostic ability of the TyG index for predicting SAP. TyG index: Triglyceride-glucose index; AUC: Area under the receiver operating characteristic (ROC) curve; CI: Confidence interval; SAP: Severe acute pancreatitis; NA: Not available.

Author	AUC	95%CI	p-value	Optimal cutoff value	Sensitivity	Specificity
Balamurli et al. [18]	0.985	0.960- 1.000	0.0005	5.39	NA	NA
Gadallah et al. [19]	0.741	NA	0.002	>5.16	76.47	72.09
Park et al. [21]	0.787	0.722- 0.851	< 0.001	4.92	92	37.4
Qin et al. [22]	0.826	NA	NA	NA	72.22	82.74
Sayiti et al. [23]	0.670	0.595- 0.745	0.001	7.74	69.5	64.6
Sieddek et al. [24]	0.827	NA	0.0006	4.94	71.4	85.1
Wang et al. [25]	0.612	0.530-0.694	< 0.05	4.823	43.1	83.2
Xinyu et al. [26]	0.637	0.533-0.741	0.014	1.003	100	21.2

Prognostic Utility of the TyG Index for ICU Admission and Mortality

Several studies extended the utility of the TyG index beyond SAP severity to include prognostic outcomes such as ICU admission and mortality. According to Park et al. [21], a TyG cutoff of 4.78 yielded an AUC of 0.650 (95% CI: 0.569-0.731; p = 0.001) for predicting ICU admission, with a sensitivity of 83.3% and specificity of 48.0%. Sayiti et al. [23] identified a higher optimal threshold of 7.68 for ICU admission (sensitivity: 68.9%, specificity: 62.7%). Regarding mortality prediction, Sayiti et al. [23] reported an AUC of 0.784 (95% CI: 0.642-0.925; p = 0.006), with a TyG cutoff of 8.40 demonstrating a sensitivity of 75.0% and specificity of 73.2%. Similarly, Qin et al. [22] observed a high prognostic value for survival in SAP patients with an AUC of 0.841, sensitivity of 90.48%, and specificity of 72.55%. These findings collectively suggest that the TyG index may serve as a valuable biomarker for identifying both disease severity and prognostic outcomes in AP, although cutoff thresholds and performance metrics vary across populations.

Discussion

To the best of our knowledge, this study represents the inaugural systematic review and meta-analysis quantitatively assessing the association between the TyG index and SAP. Our findings indicate that the TyG index is significantly elevated in patients with SAP compared to those with non-SAP, thereby suggesting its potential utility as a prognostic biomarker in clinical practice.

The TyG index, a composite biomarker derived from fasting triglyceride and glucose concentrations, has emerged as a reliable surrogate for insulin resistance (IR), providing a practical alternative to the hyperinsulinemic-euglycemic clamp, which is considered the gold standard for assessing IR [32]. Numerous studies have demonstrated that the TyG index surpasses traditional measures of IR, such as the Homeostasis Model Assessment of Insulin Resistance (HOMA-IR), in the identification of metabolic dysfunction [33, 34]. Due to its significant association with IR, the TyG index has been extensively implicated in the pathogenesis of metabolic syndrome, type 2 diabetes, cardiovascular diseases, NAFLD, and chronic kidney disease [35].

In the context of SAP, elevated values of the TyG index may indicate underlying metabolic disturbances that exacerbate pancreatic injury. Hyperglycemia is known to promote oxidative stress, enhance the release of pro-inflammatory cytokines, and exacerbate pancreatic necrosis. Concurrently, hypertriglyceridemia contributes to ischemia-reperfusion injury, microcirculatory impairment, and systemic inflammation [36, 37]. These synergistic effects plausibly elucidate the robust association observed between a high TyG index and an increased risk of SAP.

Multiple studies included in our meta-analysis substantiate this hypothesis. Park et al. [21] demonstrated that the incorporation of the TyG index into existing clinical models significantly enhanced the prediction of SAP, exceeding the performance of conventional scoring systems in both discriminatory capacity and model fit. Similarly, Qin et al. [22] identified the TyG index as an independent risk factor for SAP and complications related to AP, with follow-up data indicating a robust association with adverse clinical outcomes. Furthermore, Yimin and Jianqiang [27] reported that elevated TyG levels were significantly correlated with both SAP and the onset of systemic inflammatory response syndrome and organ failure. Additionally, Sayiti et al. [23] provided evidence that an increased TyG index was predictive not only of SAP but also of ICU admission and mortality, independent of etiology.

Subgroup analyses provided valuable insights into potential sources of heterogeneity. Although stratification by sample size and study design produced consistent results, notable geographic variability was observed. Studies conducted in India and Korea exhibited the highest MDs in TyG values between the SAP and non-SAP groups, followed by those from China and Egypt. These disparities may reflect region-specific genetic, dietary, or environmental factors that influence metabolic profiles and inflammatory responses in AP.

Sensitivity analyses confirmed the robustness of the findings, demonstrating consistent effect sizes upon the sequential exclusion of individual studies. This methodological stability underscores the reliability of the TyG index as a prognostic marker for SAP across diverse study populations and research settings.

From a pathophysiological perspective, the link between the TyG index and SAP is biologically plausible. SAP has been strongly associated with ectopic fat deposition and IR, both of which activate inflammatory pathways mediated by nuclear factor-kB, TNF-α, leptin, and interleukin-6 [9, 38-42]. These mediators may exacerbate the inflammatory cascade in AP, accelerating progression to organ failure. Although these mechanisms provide a compelling basis for the observed association, the exact pathobiology linking IR, metabolic dysregulation, and SAP warrants further elucidation.

Despite the promising results, several limitations warrant acknowledgment. First, the number of studies available for inclusion in this analysis was limited, and there was significant heterogeneity among these studies. Although efforts were made to address this heterogeneity through sensitivity analyses and subgroup analyses based on study design, geographical location, and sample size, we were unable to identify specific sources of heterogeneity, which may be attributed to the restricted number of studies. Second, a majority of the included studies (60%) were conducted in China, which may constrain the generalizability of the findings on a global scale. Third, all studies included in this review were either prospective or retrospective in nature, thereby precluding the establishment of a definitive causal relationship between the TyG index and SAP. These limitations underscore the necessity for further research to elucidate the relationship between the TyG index and SAP.

## Conclusions

In conclusion, the TyG index demonstrates promising potential as a predictive biomarker for SAP. Its simplicity, accessibility, and consistent association with SAP across diverse populations make it a valuable tool for early risk stratification. Future prospective investigations should aim to validate these findings, address inter-study heterogeneity, and further integrate the TyG index into clinical prognostic models for improved management of AP.
